# Decreased Functional Connectivities of Low-Degree Level Rich Club Organization and Caudate in Post-stroke Cognitive Impairment Based on Resting-State fMRI and Radiomics Features

**DOI:** 10.3389/fnins.2021.796530

**Published:** 2022-02-16

**Authors:** Guofu Miao, Bo Rao, Sirui Wang, Pinyan Fang, Zhuo Chen, Linglong Chen, Xin Zhang, Jun Zheng, Haibo Xu, Weijing Liao

**Affiliations:** ^1^Department of Rehabilitation Medicine, Zhongnan Hospital of Wuhan University, Wuhan, China; ^2^Department of Radiology, Zhongnan Hospital of Wuhan University, Wuhan, China; ^3^Department of Radiology, TEDA International Cardiovascular Hospital, Tianjin, China

**Keywords:** post-stroke cognitive impairment, resting-state functional magnetic resonance imaging, functional connectivity, rich club organization, graph theory

## Abstract

**Background:**

Stroke is an important cause of cognitive impairment. Rich club organization, a highly interconnected network brain core region, is closely related to cognition. We hypothesized that the disturbance of rich club organization exists in patients with post-stroke cognitive impairment (PSCI).

**Methods:**

We collected data on resting-state functional magnetic resonance imaging (rs-fMRI) with 21 healthy controls (HC), 16 hemorrhagic stroke (hPSCI), and 21 infarct stroke (iPSCI). 3D shape features and first-order statistics of stroke lesions were extracted using 3D slicer software. Additionally, we assessed cognitive function using the Montreal Cognitive Assessment (MoCA) and Mini-Mental State Examination (MMSE).

**Results:**

Normalized rich club coefficients were higher in hPSCI and iPSCI than HC at low-degree *k*-levels (*k* = 1–8 in iPSCI, *k* = 2–8 in hPSCI). Feeder and local connections were significantly decreased in PSCI patients versus HC, mainly distributed in salience network (SN), default-mode network (DMN), cerebellum network (CN), and orbitofrontal cortex (ORB), especially involving the right and left caudate with changed nodal efficiency. The feeder and local connections of significantly between-group difference were positively related to MMSE and MoCA scores, primarily distributed in the sensorimotor network (SMN) and visual network (VN) in hPSCI, SN, and DMN in iPSCI. Additionally, decreased local connections and low-degree ϕ_norm_(k) were correlated to 3D shape features and first-order statistics of stroke lesions.

**Conclusion:**

This study reveals the disrupted low-degree level rich club organization and relatively preserved functional core network in PSCI patients. Decreased feeder and local connections in cognition-related networks (DMN, SN, CN, and ORB), particularly involving the caudate nucleus, may offer insight into pathological mechanism of PSCI patients. The shape and signal features of stroke lesions may provide an essential clue for the damage of functional connectivity and the whole brain networks.

## Introduction

The stroke prevalence in China was 1,115 cases per 100,000 people in 2013, and it shows the tendency of marked increase (Wu et al., 2019). Most survivors after stroke are accompanied by different types of dysfunction. Among them, post-stroke cognitive impairment (PSCI) is a series of syndromes that meets the diagnostic criteria of cognitive impairment after stroke, containing the deficits of various cognitive domains such as memory, attention, language, executive function, and visual-spatial ability (Jokinen et al., 2015). About 30% of stroke survivors had cognitive impairment (Sun et al., 2014), which can influence the quality of life with stroke patients and increases the financial pressure of the family (Kwon et al., 2020).

Resting-state functional magnetic resonance imaging (fMRI) was used to analyze the dynamic interactions of blood oxygen level-dependent (BOLD) signal, reflected the spontaneous functional activity of neurons among brain regions (Rubinov and Sporns, 2010; Sporns, 2013), which could evaluate cognitive function (Bigler, 2014). Recently, some studies showed that the alterations in functional connectivity (FC) were closely associated with PSCI. The reduction of FCs across the cerebral hemisphere after acute ischemic stroke was correlated with impaired cognitive performance (Carter et al., 2010; Puig et al., 2018). The FC analyses of the default-mode network (DMN) were decreased after stroke (Tuladhar et al., 2013; Jiang et al., 2018), which might reveal the pathogenesis of cognitive impairment (Ding et al., 2014). A study investigated the altered FC of the hippocampal subfields belonging to DMN in stroke patients and found the decline of FC between hippocampal and inferior parietal lobule (Jung et al., 2021). Stroke patients had significant impairment in the FCs between the DMN nodes by seed-based connectivity analysis (Dacosta Aguayo et al., 2015). Our team found that the fractional amplitude of low-frequency fluctuations and seed-based FC values distributed in DMN in hPSCI and salience network (SN) in iPSCI were decreased compared with healthy volunteers (Wang et al., 2021). Interestingly, after receiving cognitive therapy, the FC in hippocampal increased in PSCI patients (Yang et al., 2014). Park et al. (2014) also found that restored DMN connectivity was relevant to the recovery of cognitive function. These studies mainly paid attention to the DMN connectivity and seed-based FC in PSCI patients. Cognitive impairment caused by stroke might be not only limited to localized brain area damage but also related to the disturbance of neural information transfer in the whole brain network (Rehme and Grefkes, 2013). However, a few studies investigated the changes in whole-brain network topology based on functional connectivity in PSCI patients.

Graph-theoretic analyses in resting-state fMRI could describe brain network topology and the characteristic of clinical syndromes and assess rich club organization of brain networks (Medaglia, 2017). Rich club organization was densely interconnected in the central hub of a network, promoting interregional neural signaling and integrated information (van den Heuvel et al., 2013). We segmented the cortical surface and cerebellum into 116 regions using the Automated Anatomical Labeling (AAL), and each brain region was defined as a node. Some core or hub brain regions are named rich club nodes, and the remaining nodes are peripheral nodes. Rich club connections, highly interconnected networks in brain core regions, could most efficiently transmit neural information in the brain network, which is vital for cognition due to directly regulating brain function across multiple isolated areas and optimizing cognitive processes (Kim and Min, 2020). Feeder connections were a core–periphery structure, integrating and transferring information from rich club and local connections. Local connections are connected between periphery regions and created a periphery–periphery structure. Rich club analysis might be helpful in understanding stroke pathophysiology, underlying diagnosis biomarker, and functional recovery (Lim and Kang, 2015). Rich club analysis has been extensively applied to cognitive impairment caused by Alzheimer’s disease (AD) (Daianu et al., 2015; Yan et al., 2018), cerebral small vessel disease (van Leijsen et al., 2019), and frontal and temporal gliomas (Liu et al., 2020). However, a few studies of rich club analysis on PSCI patients had been conducted.

We analyzed the changes in rich club organization using graph theory and assumed that the disturbance of rich club organization existed in hemorrhagic post-stroke cognitive impairment (hPSCI) and infarct post-stroke cognitive impairment (iPSCI). In addition, we explored the correlations between the clinical performance of PSCI, radiomic features of stroke lesions, and the significant alterations of FCs in patients. We hypothesized that those relationships might be different in various types of stroke.

## Materials and Methods

### Participants

Fifty-eight Chinese subjects were recruited into this study between January 2019 and December 2020. Among them, 37 cognitive impairment patients were recruited from the Department of Rehabilitation Medicine in Zhongnan Hospital, including 16 hPSCI patients and 21 iPSCI patients. Twenty-one healthy controls (HC) with matched age, sex, and education level were enrolled from the community by advertisements. In conformity with the Helsinki Declaration, the study was approved by the Medical Research Ethics Committee and Institutional Review Board of Zhongnan Hospital (Approval Number: 2019012). All participants provided written informed consent.

All subjects underwent two extensive neuropsychological tests assessed by a professional therapist, including the Beijing version of the Montreal Cognitive Assessment (MoCA) (Burton and Tyson, 2015) and the Chinese version of the Mini-Mental State Examination (MMSE) (Burton and Tyson, 2015). The inclusion criteria applied to all PSCI patients included the following: (1) the diagnosis of stroke under the Chinese National Criteria in Diagnostic Essentials of Various Cerebrovascular Diseases revised at the Fourth National Cerebrovascular Diseases Academic Conference in 1995, confirmed by CT or MRI examination (Neurology and Neurosurgery, 1996); (2) diagnosis of first-time stroke; (3) onset time ranging 7 days from 3 months in the early subacute period (Bernhardt et al., 2017); (4) age range from 40 to 80 (Jiang et al., 2018); (5) reporting mild or moderate impairment, 10 ≤ MMSE score ≤ 27 (>6 years of education) (Chua et al., 2019); (6) right-handed subjects; (7) no severe aphasia and completing the entire experiment; and (8) all participants signed an informed consent form. All patients and HC were required to meet the following exclusion criteria: (1) vital signs were unstable; (2) after craniotomy or skull defect; (3) cognitive decline caused by AD, encephalitis, traumatic brain injury, and Parkinson’s disease; (4) unable to complete neuropsychological tests for severe depression or affective disorders; and (5) contraindications of MRI scanning.

### Image Acquisition

All subjects were scanned on a MAGNETOM Trio 3.0 Tesla MR scanner (Siemens, Germany). The scanning protocol contained T1-weighted images and rs-fMRI data using the standard head coil. It was important for subjects that we used headphones and cushions to reduce the noise in scanning. All participants were required to keep their eyes closed, reduce movement, and stay awake. All T1-weighted images were acquired by a 3D magnetization-prepared rapid gradient echo (3D-MPRAGE) sequence for anatomical reference. The scanning parameters were the following: TR/TE = 2,000/2.3 ms, thickness = 1.0 mm, FA = 8°, FOV = 225 mm × 240 mm, and voxel size = 1 mm × 1 mm × 1 mm. All rs-fMRI datasets were obtained with a gradient echo-planar imaging sequence (EPI) with the following parameters: TR/TE = 2,000/30 ms, FOV = 240 mm × 240 mm, flip angle (FA) = 78°, matrix = 64 × 64, thickness = 4.0 mm, number of slices = 35, and voxel size = 3.75 mm × 3.75 mm × 4 mm. Each subject was scanned 480 s and acquired 240 time points.

### Image Segmentation

A professional radiologist manually segmented all lesion regions with the T1-weighted images in hPSCI and iPSCI patients using the 3D-Slicer software version 4.13.0^1^. 3D shape features and first-order statistics were extracted with radiomics module. Besides, another radiologist validated the above information. Intraclass correlation coefficient (ICC) was used to assess the consistency of the two radiologists. The reliability was great when the ICC value was higher than 0.75.

### Image Preprocessing

Image preprocessing was performed with the DPABI software^2^ implemented in SPM12^3^, which was run in the environment of MATLAB (Mathworks, Natick, MA, United States). First, we converted the DICOM files to NIFTI images. Second, considering that subjects need to adapt to the scanning environment for a bit of time, the first 10 volumes of BOLD sequences were discarded. Third, the effects of head motion and slice timing were corrected in the remaining volumes of BOLD sequences. Fourth, the functional images were normalized to the Montreal Neurological Institute (MNI) space by using the information of T1 structural images with a resampled voxel size of 3 mm × 3 mm × 3 mm. Fifth, the images underwent spatial smoothing using the full width at half maximum (FWHM) Gaussian kernel of 4 mm × 4 mm × 4 mm to reduce the spatial noise. Finally, the high-frequency noises of respiratory and heartbeat were eliminated by removing the linear trend of time courses and temporally band-pass filtering (0.01-0.08 Hz). Notably, the range of head motion in all subjects was no more than 2° of rotation in the x, y, or z directions, or 2 mm of displacement.

### Network Construction

This study used the AAL 116 template to segment the cortical surface and cerebellum into 116 regions (58 regions per hemisphere) as network nodes (Tzourio-Mazoyer et al., 2002). We extracted the averaged time series of the BOLD signal of each node. Then, we computed the Pearson’s correlation coefficients between a node, and the other remaining nodes acted as the edges. In this way, a 116 × 116 functional connection matrix was successfully constructed for each participant. Sparsity threshold values ranged from 0.05 to 0.5 at intervals of 0.01, which removed spurious edges (Xiao et al., 2019; Zhu et al., 2021).

### Detection of Rich Club Organization

The process of network analysis was performed using the GRETNA toolbox^4^ in the environment of MATLAB.

The degree (*k*) was defined as the number of edges that the node shared with other nodes. Rich club coefficients (ϕ(*k*)) were the density of connections between rich club nodes. The higher the rich club coefficients, the closer the connections between rich club nodes. The formula for calculating ϕ(*k*) is as follows (Ball et al., 2014; Liu et al., 2021):


(1) $$\phi \,\left(k\right)=\frac{2\,E_{>k}}{N_{>k}\,\left(N_{>k}-1\right)}$$

Among them, *N* represents the number of all nodes in a parcelation. *E* represents the number of edges. Here, *ϕ(k)* is defined as the ratio between the total actual number of edges (*E*_>_*_*k*_*) and the possible number of maximum edges that connect nodes of degree k or higher over a range of *k*-values.

As higher-degree nodes are likely to tend to be interconnected to each other, ϕ(*k*) is typically normalized. Normalized rich club coefficients (ϕ_*norm*_(*k*)) were calculated using ϕ(*k*) normalized relative to a set of 1,000 comparable random networks (ϕ_*random*_(*k*)) (Rubinov and Sporns, 2010; Yan et al., 2018). ϕ_*norm*_(*k*) was higher than 1 to indicate the existence of a rich club organization in the brain network for each participant (Daianu et al., 2015). The formula for calculating ϕ_*norm*_(*k*) is as follows (van den Heuvel and Sporns, 2011):


(2) $$\phi_{n\,o\,r\,m}\,\left(k\right)=\frac{\phi \,\left(k\right)}{\phi_{r\,a\,n\,d\,o\,m}\,\left(k\right)}$$

According to the classification of network nodes into the rich club and peripheral regions, the network edges were classified into local connections, connecting between peripheral nodes; feeder connections, connecting one rich club node with one peripheral node, and rich club connections, connecting between rich club nodes.

### Statistical Analysis

IBM SPSS performed the statistical analysis for version 23^5^. Mean ± standard deviation represents the numerical variables. We used the Shapiro–Wilk (S–W) test to analyze the normal distribution. Group differences were tested using the chi-square test in sex, lesion side, number of lesions and lesion location, and one-way analysis of variance (ANOVA) in age, years of education, MMSE, and MoCA score. Two-sample t-test was used to test the difference in stroke duration and lesion volume in hPSCI and iPSCI.

We used ANOVA to evaluate group differences in continuous variables of connectivity strength, nodal efficiency, and ϕ_norm_(k). Then two-sample t-tests (HC group compared with patient groups), acting as *post hoc* tests, were conducted, with FDR corrected to the *p*-values to correct for multiple comparisons (Yan et al., 2018). A value of *p* < 0.05 represented significant difference.

Pearson’s correlations were used to assess the relationship between the clinical performance of cognitive impairment, radiomic features, and FC in each group.

## Results

### Demographic and Clinical Data

This study recruited 16 hPSCI, 21 iPSCI, and 21 HC. Table 1 shows the demographic and clinical information for all participants. There were no significant group differences for sex, age, and years of education. The stroke duration, lesion side, and number of lesions were not significantly different in hPSCI and iPSCI patients. Significant group differences were found in MMSE and MoCA scores. The patients with hPSCI and iPSCI performed significantly worse than HC on cognitive function, and there was no significant difference between hPSCI and iPSCI. The lesion location in hPSCI patients was principally concentrated on the thalamus and basal ganglia, while iPSCI patients occurred with cortical infarction (*p* = 0.003). The lesion volume in iPSCI patients was higher than that in hPSCI patients (*p* = 0.004).

**TABLE 1:** Baseline of demographic and clinical characteristics.

Group	HC	hPSCI	iPSCI	p
	*N* = 21	*N* = 16	N = 21	
Sex (male/female)	17/4	12/4	18/3	0.568
Age (years)	60.10 ± 6.59	60.38 ± 9.78	55.81 ± 10.61	0.216
Lesion side (left/right)	-	10/6	16/5	0.475
Number of lesions (single/multiple)	-	12/4	9/12	0.093
Lesion location (cortex/subcortex)	-	3/13	15/6 **^‡^**	0.003
Lesion volume (voxel)	-	25,027 ± 32,775	83,149 ± 69,981 **^‡^**	0.004
Education (years)	11.81 ± 2.80	11.31 ± 3.22	12.24 ± 3.03	0.652
Stroke duration (days)	-	49.93 ± 22.80	47.57 ± 50.00	0.866
MoCA	29.10 ± 1.14	16.62 ± 4.57 *	15.48 ± 5.22 **^†^**	< 0.001
MMSE	29.33 ± 0.73	20.69 ± 4.66 *	18.57 ± 5.58 **^†^**	< 0.001

Values for age, education, MoCA, and MMSE were derived from ANOVA with post hoc test. Sex, lesion side, number of lesions, and lesion location were derived from chi-square test. Two-sample t-tests were used for lesion volume and stroke duration. *Compared with the HC group, p < 0.001 in the hPSCI group; **^†^**compared with the HC group, p < 0.001 in the iPSCI group. **^‡^**Compared with the hPSCI group, p < 0.05 in the iPSCI group. HC, healthy controls; hPSCI, hemorrhagic post-stroke cognitive impairment; iPSCI. infarct post-stroke cognitive impairment; MMSE, Mini-Mental State Examination; MoCA, Montreal Cognitive Assessment; ANOVA, one-way analysis of variance.

### Rich Club Nodes of All Groups

**FIGURE 1:**
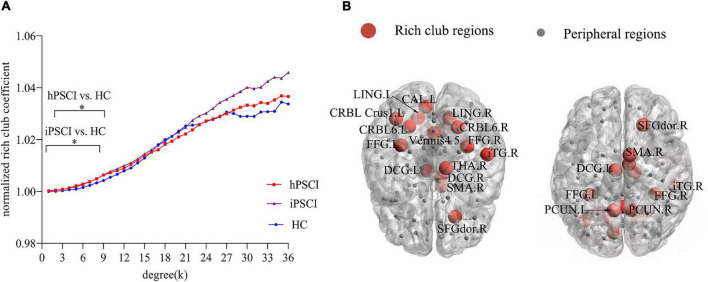
Rich club organization. **(A)** Normalized rich club coefficients at k value ranging from 1 to 36. *The differences were seen in hPSCI (*k* = 2-8) and iPSCI (*k* = 1-8) versus HC (*p* < 0.05). **(B)** The number of rich club regions (red nodes) in all participants. HC, healthy controls; hPSCI, hemorrhagic post-stroke cognitive impairment; iPSCI, infarct post-stroke cognitive impairment.

Rich club organization existed in the brain network in all groups when ϕ_norm_(k) was greater than 1 (Figure 1A). Most subjects (90%) were found to have the rich club effects at degree levels (k) (Daianu et al., 2015; Yan et al., 2018). We selected the top 17 (15%) highest-degree nodes to represent rich club nodes based on the averaged nodal degree across all groups (Figure 1B). The identified rich club nodes comprised the following: the left and right precuneus, left and right fusiform, left and right lingual, right inferior temporal gyrus, left and right cerebellum_6, left and right middle cingulum, right superior frontal gyrus, left cerebellum_1, right supplementary motor area, left calcarine, and vermis_4, 5, right thalamus. The rest of the nodes were defined as peripheral nodes.

### The Disturbance of Rich Club Organization

The ϕ_norm_(k) was higher than 1 at *k* value ranging from 1 to 36. ϕ_norm_(k) was significantly higher in hPSCI and iPSCI patients than HC at low-degree *k*-levels, detailedly *k* = 1–8 in the iPSCI group and *k* = 2–8 in the hPSCI group (Figure 1A).

**FIGURE 2:**
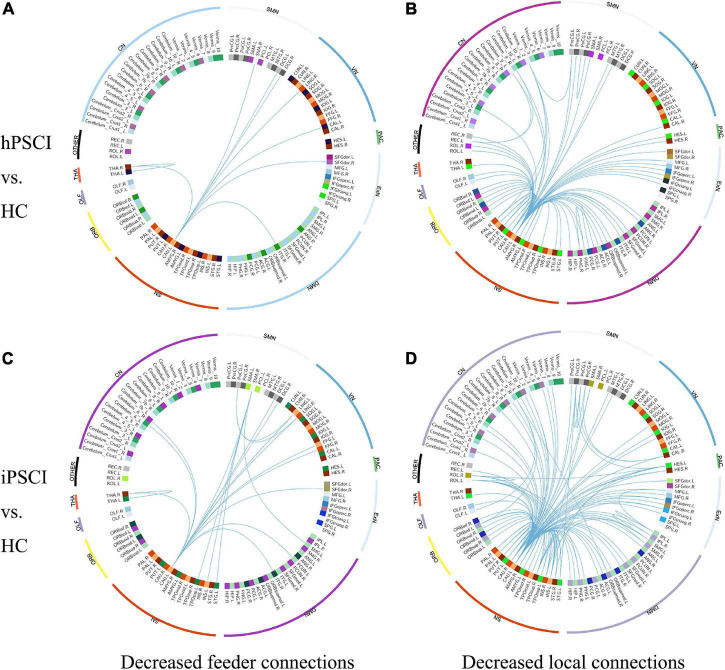
The decrease in functional connectivity in PSCI relative to HC. **(A)** Decreased feeder connections in hPSCI versus HC. **(B)** Decreased local connections in hPSCI versus HC. **(C)** Decreased feeder connections in iPSCI versus HC. **(D)** Decreased local connections in iPSCI versus HC. HC, healthy controls; hPSCI, hemorrhagic post-stroke cognitive impairment; iPSCI, infarct post-stroke cognitive impairment; ORB, orbitofrontal cortex; SN, salience network; DMN, default-mode network; ExN, external frontoparietal network; PAC, primary auditory cortex; VN, visual network; SMN, sensorimotor network; CN, cerebellum network; THA, thalamus; OLF, Olfactory cortex.

We found that rich club connections had no significant differences in PSCI patients relative to HC. FCs were lower in hPSCI patients than HC with nine feeder connections (Figure 2A and Supplementary Table 1) and 71 local connections (Figure 2B and Supplementary Table 2), which was mainly distributed in the SN, DMN, cerebellum network (CN), and orbitofrontal cortex (ORB). FCs were significantly decreased in iPSCI patients versus HC with 20 feeder connections (Figure 2C and Supplementary Table 3) and 117 local connections (Figure 2D and Supplementary Table 4), primarily distributed in the SN, DMN, CN, and ORB.

### Whole-Brain Functional Connectivity of Abnormal Nodes

Some nodes of significantly decreased FCs existed in hPSCI and iPSCI versus HC, which might be essential nodes leading to reduced communication. We identified two abnormal nodes with highest edges of aberrant connections according to plotting the frequency of aberrant connections linked by the 116 nodes in hPSCI and iPSCI groups.

**FIGURE 3:**
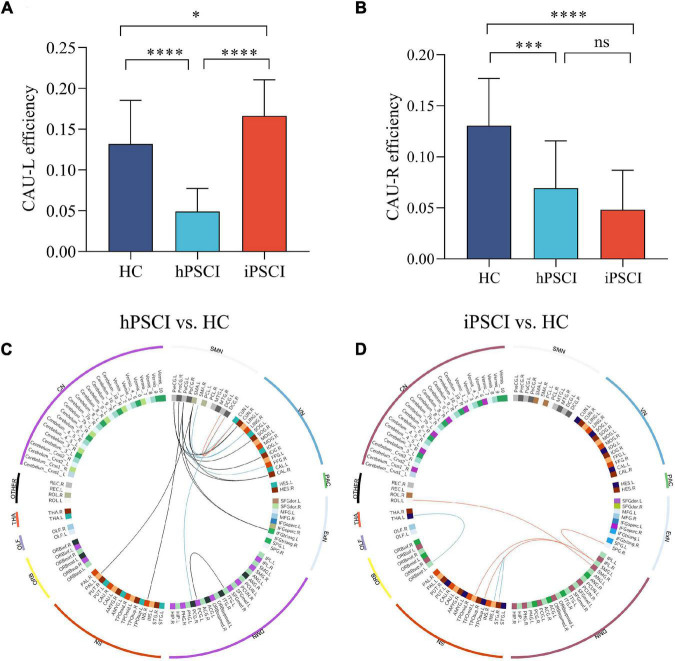
The alteration in nodal efficiency in PSCI versus HC. **(A)** CAU_L efficiency. **(B)** CAU_R efficiency. Ns, not significant; *p < 0.05; ^***^p < 0.001; ^****^p < 0.0001. The positive correlation between the decrease in functional connectivity and clinical performance. **(C)** Decreased feeder connections were positively correlated to MMSE (red lines) and MoCA (blue lines), and decreased local connections were positively correlated to MoCA (black lines) in hPSCI patients versus HC. **(D)** Decreased local connections were positively correlated to MMSE (red lines) and MoCA (blue lines) in iPSCI patients versus HC. CAU_L, left caudate; CAU_R, right caudate; HC, healthy controls; hPSCI, hemorrhagic post-stroke cognitive impairment; iPSCI, infarct post-stroke cognitive impairment; MoCA, Montreal Cognitive Assessment; MMSE, Mini-Mental State Examination; ORB, orbitofrontal cortex; SN, salience network; DMN, default-mode network; ExN, external frontoparietal network; PAC, primary auditory cortex; VN, visual network; SMN, sensorimotor network; CN, cerebellum network; THA, thalamus; OLF, olfactory cortex.

Compared with HC, two peripheral nodes with the most declined connections were found in hPSCI, including left caudate (CAU_L) with 31 abnormal edges and right caudate (CAU_R) with 4 abnormal edges. Two peripheral nodes with the most declined connections were found in iPSCI, including CAU_L with 43 abnormal edges and CAU_R with 5 abnormal edges. Group differences were seen in CAU_L efficiency [F (2,55) = 32.51, *p* < 0.001] and CAU_R efficiency [F (2,55) = 19.94, *p* < 0.001]. A significant decrease in CAU_L efficiency was observed in hPSCI versus HC (*p* < 0.001) (Figure 3A). CAU_L efficiency was lower in hPSCI than in iPSCI (*p* < 0.001) (Figure 3A). CAU_L efficiency was significantly increased in iPSCI patients versus HC (*p* = 0.0382) (Figure 3A). Compared with HC, the significant declines in CAU_R efficiency were seen in hPSCI (*p* = 0.0003) and iPSCI groups (*p* < 0.001) (Figure 3B).

### Behavioral Correlation Analysis

We first found that some FCs had significant differences between the two patient groups and HC. Then these FCs were positively correlated with the MMSE and MoCA scores, chiefly distributed in the sensorimotor network (SMN) and visual network (VN) in hPSCI (Figure 3C and Supplementary Table 5), SN, and DMN in iPSCI (Figure 3D and Supplementary Table 6).

### Radiomic Feature Correlation Analysis

**FIGURE 4:**
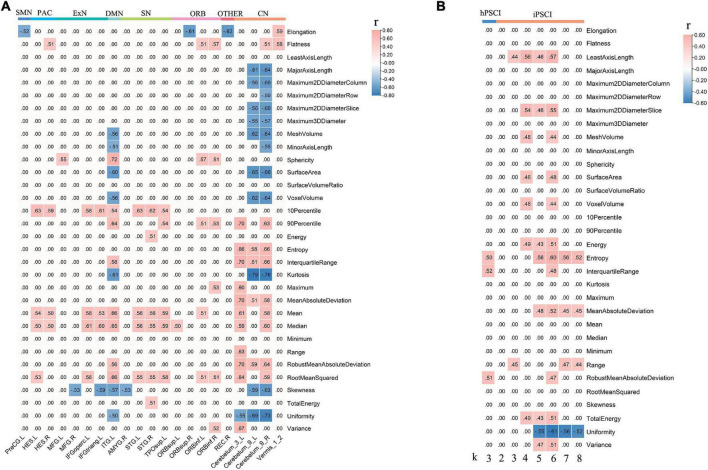
The correlation between decreased FCs linked to CAU_L, ϕ_norm_(k), and radiomic features. **(A)** Decreased local connections were correlated with radiomic features in hPSCI. **(B)** ϕ_norm_(k) was correlated with radiomic features in hPSCI and iPSCI. CAU_L, left caudate; hPSCI, hemorrhagic post-stroke cognitive impairment; hPSCI, infarct post-stroke cognitive impairment; ORB, orbitofrontal cortex; SN, salience network; DMN, default-mode network; ExN, external frontoparietal network; PAC, primary auditory cortex; VN, visual network; SMN, sensorimotor network; CN, cerebellum network; FC, functional connectivity.

**FIGURE 5:**
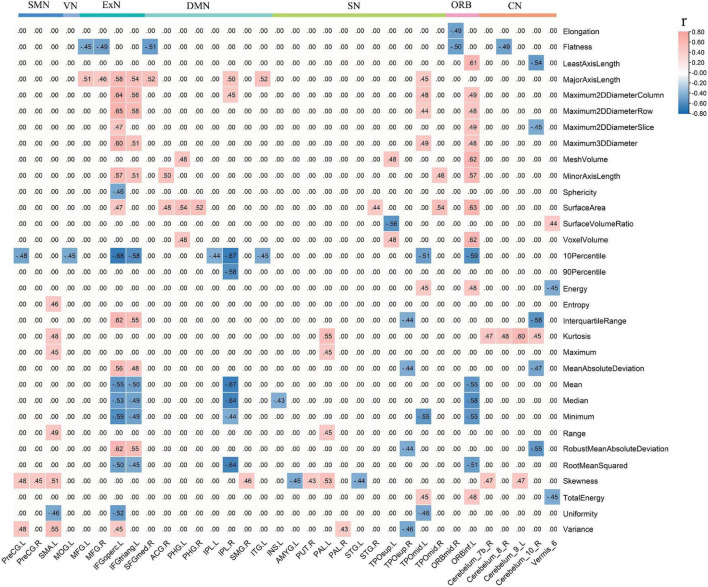
Decreased local connections linked to CAU_L were correlated with radiomic features in iPSCI. CAU_L, left caudate; iPSCI, infarct post-stroke cognitive impairment; SMN, sensorimotor network; VN, visual network; ExN, external frontoparietal network; DMN, default-mode network; SN, salience network; ORB, orbitofrontal cortex; CN, cerebellum network.

The ICC value was [(0.81 ± 0.037), *p* < 0.05], representing great consistency for the segmentation of hemorrhage and infraction lesions. The FCs linked to CAU_L and radiomic features were performed with correlation analysis. We found the negative correlation between decreased local connections and most 3D shape features in hPSCI patients, and a positive correlation in most first-order statistics (Figure 4A and Supplementary Table 7). There was a positive correlation between local connections and most 3D shape features, and a negative correlation in most first-order statistics in iPSCI patients (Figure 5 and Supplementary Table 9). Besides, ϕ_norm_(k) in hPSCI patients (*k* = 3) and iPSCI patients (*k* = 2–8) were positively correlated to most 3D shape features and first-order statistics (Figure 4B and Supplementary Table 8).

## Discussion

In this study, we used a graph-theoretical approach to analyze the FCs of whole brain in healthy volunteers, the patients of iPSCI and hPSCI. Our study had some findings, which are as follows: (1) ϕ_norm_(k) was higher at low-degree k-levels in patients than HC. (2) The undisturbed rich club connections were relatively preserved in patients with PSCI. PSCI tended to affect the feeder and local connections of the SN, DMN, CN, and ORB, especially involving the caudate nucleus (CAU) belonging to the SN. (3) Aberrant CAU efficiencies were observed in the hPSCI and iPSCI groups. (4) Decreased connections involving SMN and VN in hPSCI, and the ones involving SN and DMN in iPSCI, were positively related to the MMSE and MoCA scores. (5) 3D shape features and first-order statistics were correlated with decreased local connections and low-degree ϕ_norm_(k) in patients.

### The Changes in Normalized Rich Club Coefficients

We observed that ϕ_norm_(k) increased in the lower-degree regions (*k* = 1-8 in iPSCI and *k* = 2-8 in hPSCI) compared with the HC group. Our result indicated that rich club nodes in low-degree regions were more likely to be connected to peripheral nodes, which suggested a communication relay to integrate global information by forming locally clustered and segregated communities of the connectome (Cao et al., 2020). Besides, the higher-degree regions found no significant difference in patients, implying that the high-degree k-level regions remain as highly interconnected nodes (Daianu et al., 2016). With the value of k degree increased, the main core regions of the brain network were maintained by peeling off low-degree nodes. In our study, most patients with PSCI were mild and moderate, which might only damage some regions at lower-degree (peripheral nodes). Daianu et al. (2015) considered that ϕ_norm_(k) was a potential metric to detect the network differences, and found that ϕ_norm_(k) was increased within low-degree k-levels rather than high-degree in AD and mild cognitive impairment (MCI) patients versus HC, which was consistent with our result. The brain connectivity of peripheral regions may be disrupted rather than rich club connections in patients with mild and moderate cognitive impairment after stroke.

### Changes of Rich Club, Feeder, and Local Connections

We found no significant difference in rich club connections, which indicated that rich club connections were relatively stable in hPSCI and iPSCI, which was in accordance with the finding of ϕ_norm_(k). Rich club connections played an essential role in the integrative brain processes and formed a central backbone for global brain communication, whose interruption severely impacted the whole brain (van den Heuvel et al., 2013). A study found that rich club connections kept stable in patients with subjective cognitive decline (Daianu et al., 2015). Our study showed that the relatively stable rich club connections in patients with mild and moderate PSCI suggested the retention of partial cognitive function, similar to the damaged pattern of subjective cognitive decline patients.

Our result showed that feeder and local connections were declined in hPSCI and iPSCI patients. Feeder connections could create a core–periphery structure, transferring information transmission based on network among these nodes, integrating and receiving information from various peripheral regions (Liu et al., 2021). Our result indicated that the ability of received and integrated information was declined. Local connections were the periphery–periphery structure by linking non-rich club nodes to non-rich club nodes (van den Heuvel et al., 2013). We observed the descend of the transferred information in local connections. Notably, the number of damaged local connections was much greater than that of feeder connections, which manifested that mild and moderate PSCI might mainly damage local connections. Because peripheral regions reduced persistence and lower levels in the hierarchical network, local and feeder connections were easier to damage (Yan et al., 2018). Cao et al. (2020) found that the decline in feeder and local connections were main factors leading to the lower efficiency of brain network in MCI and AD, which was in accord with this study. Thus, we speculated that the stroke events might disturb the ability to receive and integrate information of feeder and local connections, eventually causing cognitive impairment.

Additionally, we detected that descended feeder and local connections were majorly distributed in the SN, DMN, CN, and ORB in both patient groups. When individuals were concentrated on the internal tasks rather than the external environment, DMN preferentially was active, and SN was an essential network in the dynamic switching between DMN and central executive network (CEN) (Goulden et al., 2014). The SN and DMN played crucial roles in various cognitive domains (Ding et al., 2014; Goulden et al., 2014). Chand et al. (2017) confirmed that the damaged interaction between DMN, CEN, and SN was closely related to MCI. Interestingly, we also found that CN connectivity was declined in patients. The output channel from the cerebellum comprises substantial projections to relevant cerebral regions, such as the prefrontal cortex (Buckner, 2013). The cerebellum could form extensive neural circuits between the cerebrum and cerebellum to modulate cognitive function. Wang et al. (2019) showed that cognitive impairment after cerebellar stroke might be related to interrupted brain network. Besides, the connections of neurons in ORB were able to integrate some signals from the external world and internal states to output the thalamus and prefrontal cortex, ongoing cognitive processing (Rudebeck and Rich, 2018). Wang et al. (2020) found that the alteration in sulcogyral patterns in the orbitofrontal cortex occurred in MCI patients. These previous studies were similar to our results. The disturbance of feeder and local connections was primarily distributed in the SN, DMN, CN, and ORB, which may be an important factor leading to cognitive impairment after stroke.

Furthermore, although there was no statistical difference in FCs and clinical performance between the two patient groups, MMSE and MoCA scores were higher in hPSCI than in iPSCI patients. The number of damaged feeder and local connections was greater in iPSCI than in hPSCI, which might be associated with the severity of cognitive impairment, consistent with the result of neuropsychological tests.

### Aberrant Connections of Caudate Nucleus

In this study, the CAU was a primary peripheral region of decreased FCs in both patient groups. Besides, the connections of CAU with other nodes were widely disturbed. CAU was an essential part of SN, which was involved in integrating global brain information (Stocco et al., 2012) and cooperated with the hippocampus to enhance memory performance (Müller et al., 2018). The damage in CAU, through cortical–caudate–cortical loops, may influence cognitive functions (Fuh and Wang, 1995). Yan et al. (2018) found that CAU_L and CAU_R contained widely aberrant connections in subjective cognitive decline patients, which was supported by our study.

Our study showed that CAU_L and CAU_R efficiencies were significantly declined in hPSCI patients versus HC. In this study, the lesion location in hPSCI patients was principally the thalamus and basal ganglia. CAU was a part of the basal ganglia. A study showed that the alteration in effective connectivity network in the prefrontal–basal ganglia circuit influenced cognitive function in patients with subcortical stoke (Zhang et al., 2020). Remarkably, PSCI patients with subcortical injury had less extra path from the left ventral anterior nucleus in the thalamus to CAU connection (Zhang et al., 2020). Therefore, we inferred that the decrease in CAU efficiencies might be related to the damage of the prefrontal–basal ganglia circuit, which was similar to the above previous study.

We found that CAU_L efficiency was increased, and CAU_R efficiency was decreased in iPSCI. In this study, iPSCI patients mainly had cortical infarction, and the CAU was not damaged. We inferred that iPSCI patients might have the compensatory phenomenon, improving nodal efficiency by reducing path length to relieve the onset of clinical symptoms. However, due to the severe brain damage, the clinical symptoms of cognitive impairment still appeared. The functional reorganization of CAU_L might underlie compensatory plasticity mechanisms (Pini et al., 2020; Zhang et al., 2021). However, CAU_R efficiency decreased in iPSCI. The hemispheric lateralization might be an essential factor to modulate cognitive specialization (Caeyenberghs and Leemans, 2014). A possible reason was that CAU was a region of leftward asymmetry according to AAL atlas (Caeyenberghs and Leemans, 2014), which might explain the differences in nodal efficiency.

### Behavioral Correlation

Our study found the FCs of significant between-group differences in SN and DMN in iPSCI patients, which was positively related to MMSE and MoCA scores. Most iPSCI patients had multiple cortical infarctions. The interruption of SN connectivity was related to the decline in cognitive function in elderly people (He et al., 2014). A study found that the FC involved in DMN was decreased in infarct stroke patients to account for the occurrence of PSCI (Tuladhar et al., 2013). We showed that the impairment of DMN and SN connectivity might be closely related to overall cognition in iPSCI, which corresponds to the above previous studies.

Our result showed that the FCs of significant between-group differences in SMN and VN in hPSCI were positively correlated with MMSE and MoCA scores. In this study, the thalamus and basal ganglia were damaged in most hPSCI patients. The thalamus and basal ganglia played roles in motor and cognition through cortico-basal ganglia-thalamo-cortical loops (Lanciego et al., 2012). The output of basal ganglia targeted the motor cortex, prefrontal cortex, and the frontal eye field, implying that damaged basal ganglia produced not only the alteration in motor function but also the aberration of cognitive impairment (Middleton and Strick, 2000). As an important relayed nucleus, the thalamus was involved in the transmission of motor pathways and cognitive processes (Rikhye et al., 2018). Thalamus was a first-order subcortical nucleus along the retina–cortical pathway, transmitting peripheral signals to the visual cortex (Saalmann and Kastner, 2011). The disturbance of the brain network caused by stroke not only existed in the vicinity of the lesion but also occurred in remotely unaffected brain areas (Rehme and Grefkes, 2013). Our results suggested that the SMN and VN related to motor and cognition might be impaired. A study found that the difference of FCs in early sensory networks, especially involving SMN and VN, was relevant to the difference in age-related cognitive performance (Stumme et al., 2020), which was the same as our results. The cognitive impairment caused by the thalamus and basal ganglia stroke was not only related to the damage of the local brain structure but also might be affected by the interference of the brain network mainly in SMN and VN.

### Radiomic Feature Correlation

In this study, we found the negative correlation between decreased local connections (CN linked to CAU_L) and 3D shape features, suggesting that the size of hemorrhagic lesions might affect the interruption of the brain network. Most of the infarct lesions were located in the blood supply area of the middle cerebral artery, consistent with the affected cortical network (distributed in DMN, ExN, and ORB). Decreased FCs were positively related to 3D shape features in iPSCI patients, which implied that the larger infarct lesions might be beneficial for repairing decreased FCs because the larger the edema and penumbra, more active neurons may remain.

First-order statistics was derived from statistical moments of the image intensity histogram, which described the distribution of voxel intensity (Yip and Aerts, 2016). The decreased local connections (distributed in CN, SN, ExN, and DMN) were positively correlated to first-order statistics in hPSCI patients, which indicated that the signal loss caused by hemosiderin deposition might damage FCs in the brain network. Infarct regions finally became glial scars with low signal through necrosis, liquefaction, absorption, and repair. We found a negative correlation between decreased local connections (distributed in DMN, ExN, and ORB) and first-order statistics in iPSCI patients, which suggested that the repair of brain tissue might restore the part of FCs in the brain network. Additionally, ϕ_norm_(k) was positively correlated with 3D shape features and first-order statistics in hPSCI (*k* = 3) and iPSCI (*k* = 2–7) patients, indicating that the features of lesions might influence the FCs in low-degree k-level.

## Limitations

There were some limitations in the current study. First, this study lacked a control group of non-cognitive impairment after stroke. Second, we only collected the data on the overall cognitive function of two cognitive scales, lacking the analysis of specific cognitive domains, such as attention, memory, and executive function. Third, considering that the sample size may be small, we did not compare the difference between mild and severe cognitive impairment in PSCI patients. Finally, future study should investigate the topological properties in the varying degrees of cognitive impairment and analyze the difference of cognitive impairment caused by the subcortical and cortical lesions.

## Conclusion

We find that rich club connections are not damaged in PSCI patients. However, the disturbances in feeder and local connections are more severe in hPSCI and iPSCI than HC, primarily distributed in cognition-related networks (SN, DMN, CN, and ORB). Rich club analysis reveals that the decrease in FCs principally focuses on CAU belonging to SN. The abnormal FCs in brain networks (SMN and VN in hPSCI, SN, and DMN in iPSCI) may be related to cognitive performance. The radiomic features of stroke lesions may imply the damage of FCs and low-degree rich club organization. These findings are helpful for further understanding of the pathogenesis with PSCI.

## Data Availability Statement

The original contributions presented in the study are included in the article/Supplementary Material, further inquiries can be directed to the corresponding author/s.

## Ethics Statement

The studies involving human participants were reviewed and approved by the Medical Research Ethics Committee and Institutional Review Board of Zhongnan Hospital. The patients/participants provided their written informed consent to participate in this study.

## Author Contributions

GM contributed to the writing of the original draft, conceptualization, methodology, and performed the formal analysis. BR contributed to the conceptualization, performed formal analysis, and wrote, reviewed, and edited the draft. SW wrote, reviewed, and edited the draft and conducted the formal analysis. PF, ZC, XZ, and JZ conducted the investigation. LC handled the visualization. HX and WL handled the supervision and funding acquisition. All authors contributed to the article and approved the submitted version.

## Conflict of Interest

The authors declare that the research was conducted in the absence of any commercial or financial relationships that could be construed as a potential conflict of interest.

## Publisher’s Note

All claims expressed in this article are solely those of the authors and do not necessarily represent those of their affiliated organizations, or those of the publisher, the editors and the reviewers. Any product that may be evaluated in this article, or claim that may be made by its manufacturer, is not guaranteed or endorsed by the publisher.

## Footnotes

1
http://www.slicer.org

2
http://rfmri.org/dpabi

3
http://www.fil.ion.ucl.ac.uk/spm

4
http://www.nitrc.org/projects/gretna/

5
http://www.spss.com/
