# Supplementary material for: Decreased Functional Connectivities of Low-Degree Level Rich Club Organization and Caudate in Post-stroke Cognitive Impairment Based on Resting-State fMRI and Radiomics Features
Source: Front Neurosci. 2022 Feb 16;15:796530. doi: 10.3389/fnins.2021.796530 (PMC8890030; doi:10.3389/fnins.2021.796530)
Supplement: Supplementary file 10 [file Data_Sheet_1.DOCX]

**Supplementary material**

**Results**

**The disturbance of rich club organization**

Table S1. The feeder connections of some brain regions were significantly decreased using two-sample t-test with FDR corrected to the P values in hPSCI patients versus HC (P < 0.0001). HC: healthy control; hPSCI: hemorrhagic post-stroke cognitive impairment.

Table S2. The local connections of some brain regions were significantly decreased using two-sample t-test with FDR corrected to the P values in hPSCI patients versus HC (P < 0.0001). HC: healthy control; hPSCI: hemorrhagic post-stroke cognitive impairment.

Table S3. The feeder connections of some brain regions were significantly decreased using two-sample t-test with FDR corrected to the P values in iPSCI patients versus HC (P < 0.0001). HC: healthy control; iPSCI: infarct post-stroke cognitive impairment.

Table S4. The local connections of some brain regions were significantly decreased using two-sample t-test with FDR corrected to the P values in iPSCI patients versus HC (P < 0.0001). HC: healthy control; iPSCI: infarct post-stroke cognitive impairment.

Table S5. Pearson’s correlations between decreased feeder, local connections and clinical performance (MMSE and MoCA scores) in hPSCI patients. The feeder, local connections were positively correlated to MMSE and MoCA scores (r>0.8, P < 0.0001). hPSCI: hemorrhagic post-stroke cognitive impairment; MoCA: Montreal Cognitive Assessment; MMSE: Mini-Mental State Examination.

Table S6. Pearson’s correlations between decreased local connections and clinical performance (MMSE and MoCA scores) in iPSCI patients. The local connections were positively correlated to MMSE and MoCA scores (r>0.7, P < 0.0001). iPSCI: infarct post-stroke cognitive impairment; MoCA: Montreal Cognitive Assessment; MMSE: Mini-Mental State Examination.

Table S7. Pearson’s correlations between decreased local connections linked to CAU_L and radiomic features (3D shape features and first-order statistics) in hPSCI patients. hPSCI: hemorrhagic post-stroke cognitive impairment; CAU_L: left caudate nucleus.

Table S8. Pearson’s correlations between $\phi_{\mathrm{norm}}(k)$ of significantly between-group difference and radiomic features (3D shape features and first-order statistics) in hPSCI and iPSCI patients. hPSCI: hemorrhagic post-stroke cognitive impairment; iPSCI: infarct post-stroke cognitive impairment.

Table S9. Pearson’s correlations between decreased local connections linked to CAU_L and radiomic features (3D shape features and first-order statistics) in iPSCI patients. iPSCI: infarct post-stroke cognitive impairment; CAU_L: left caudate nucleus.
